# Comparison of nanoparticular hydroxyapatite pastes of different particle content and size in a novel scapula defect model

**DOI:** 10.1038/srep43425

**Published:** 2017-02-24

**Authors:** Veronika Hruschka, Stefan Tangl, Yulia Ryabenkova, Patrick Heimel, Dirk Barnewitz, Günter Möbus, Claudia Keibl, James Ferguson, Paulo Quadros, Cheryl Miller, Rebecca Goodchild, Wayne Austin, Heinz Redl, Thomas Nau

**Affiliations:** 1 Ludwig Boltzmann Institute for Experimental and Clinical Traumatology, AUVA Research Centre, Vienna, Austria; 2 Austrian Cluster for Tissue Regeneration, Vienna, Austria; 3 Karl Donath Laboratory for Hard Tissue and Biomaterial Research, Department of Oral Surgery, Medical University of Vienna, Vienna, Austria; 4 Department of Materials Science and Engineering, The University of Sheffield, Sheffield, United Kingdom; 5 Research Center for Medical Technology and Biotechnology, Bad Langensalza, Germany; 6 Fluidinova S.A., Moreira da Maia, Portugal; 7 The School of Clinical Dentistry, University of Sheffield, Sheffield, United Kingdom; 8 Ceramisys Ltd., Sheffield, United Kingdom

## Abstract

Nanocrystalline hydroxyapatite (HA) has good biocompatibility and the potential to support bone formation. It represents a promising alternative to autologous bone grafting, which is considered the current gold standard for the treatment of low weight bearing bone defects. The purpose of this study was to compare three bone substitute pastes of different HA content and particle size with autologous bone and empty defects, at two time points (6 and 12 months) in an ovine scapula drillhole model using micro-CT, histology and histomorphometry evaluation. The nHA-LC (38% HA content) paste supported bone formation with a high defect bridging-rate. Compared to nHA-LC, Ostim^®^ (35% HA content) showed less and smaller particle agglomerates but also a reduced defect bridging-rate due to its fast degradation The highly concentrated nHA-HC paste (48% HA content) formed oversized particle agglomerates which supported the defect bridging but left little space for bone formation in the defect site. Interestingly, the gold standard treatment of the defect site with autologous bone tissue did not improve bone formation or defect bridging compared to the empty control. We concluded that the material resorption and bone formation was highly impacted by the particle-specific agglomeration behaviour in this study.

Autologous bone graft is considered the gold standard for the treatment of low weight-bearing bone defects. Autologous bone grafts have osteogenic, osteoinductive and osteoconductive properties while being non immunogenic. However, complications at the graft harvesting site, including infection, prolonged wound drainage, large hematomas, vascular injuries, the need for revision surgery, pain, sensory loss, herniation, fracture and cosmetically problematic scars may occur^1^,^2^,^3^,^4^,^5^,^6^,^7^. Synthetic substitutes, such as hydroxyapatite (HA) or tricalcium phosphate based materials, also possess osteoconductive properties and present an alternative to autologous bone grafts. Nanocrystalline HA, for example the commercially available paste Ostim^®^, has been successfully used in the fields of oral, maxillofacial, and orthopaedic surgery, showing good biocompatibility and the potential to support bone formation without eliciting an inflammatory response^8^,^9^,^10^,^11^,^12^,^13^,^14^. As an injectable paste, Ostim^®^ can be applied in a minimally invasive manner to completely fill irregular defects. Laschke *et al*. reported that the degradation process of Ostim^®^ enabled cellular migration into the augmentation material and thereby supported vascularisation and facilitated bone formation^14^. Brandt *et al*. observed agglomerations of the Ostim^®^ nanocrystals that became more dense over time, remaining embedded in bone and connective tissue without further resorption^11^. While single nanoparticles might provoke inflammatory responses and inhibitory effects^15^,^16^, agglomerates increased the fibronectin adsorption which influences osteoblast adherence^17^,^18^,^19^. The surface topography of HA plays a critical role in bone regeneration. HA crystals in hard tissues have been reported to be nanoscale rod-like or plate-like crystals^20^. Nanostructured surfaces of implant materials have been shown to enhance cellular activity and induce bone formation^14^,^16^,^21^,^22^,^23^,^24^. We hypothesize that the HA content and particle size of the nanocrystalline HA paste may affect material behaviour with regard to resorption and bone formation and therefore the *in vivo* outcome. In our study, three HA pastes differing in HA content and particle sizes were evaluated. Based on preliminary *in vitro* studies^25^, the following pastes were selected: a paste with a concentration of HA of 38% and rod shaped particles with a mean length of 40–60 nm (nHA-LC), a paste with a HA content of 48% and a similar mean particle length of 40–60 nm with a more elongated particle shape (nHA-HC) and the commercially available Ostim^®^ with a HA content of 35%, rod shaped particles with reported average dimensions of 150 nm × 25 nm^26^.

The aim of this study was to compare the potential of two newly developed bone substitute materials to facilitate bone formation, with Ostim^®^, a commercially available product, and the “gold standard” autologous cancellous bone, in an ovine defect model.

## Results

### Materials characterisation

X-ray powder diffraction (XRPD) was employed in order to evaluate the phase purity of the test materials. A full match with HA was obtained (full match with the JCPDS card 9–432) with no other phases detected (see Supplementary Fig. 1). The broad peak reflections of the patterns obtained for both nHA-LC and nHA-HC are characteristic of poorly crystalline phases and/or small crystallite size. XRD analysis was not performed on the Ostim^®^ material as the information was readily available from the supplier and stated that it is a phase-pure hydroxyapatite. Thus, we can conclude that both commercially available and in house materials do not contain any other phase and appear to be stoichiometric hydroxyapatites.

Thermogravimetric analysis was performed in order to quantify water content of the non-commercial pastes (see Supplementary Fig. 2). A rapid weight loss up to the temperatures of *ca.* 400 °C was observed for both pastes characteristic of the water release from the surface and lattices of hydroxyapatite particles^27^. After this temperature no more weight loss was detected. Thus, it was possible to estimate the amount of water present in the materials at 38 wt% for nHA-LC and 48 wt% for nHA-HC, with the former comparable to that of Ostim^®^ (35 wt%) and the latter having the highest ceramic loading among all the materials tested. Moreover, no further weight loss was detected after all water had been removed. This shows that materials are thermally stable in the range of temperatures tested, which can be used as further (indirect) evidence of the materials stoichiometry and phase purity^28^.

Transmission electron microscopy was used to visualise the materials constituent particles at the nano-level and estimate their particle size (see Supplementary Fig. 3). Surprisingly, Ostim^®^ did not appear to consist of needle shaped nano-particles of 150 × 25 nm, as reported earlier in literature^14^,^26^. On the contrary, it comprised of two different particle types: (a) rod shaped ones with the dimensions of *ca.* 70 × 20 nm and (b) interconnected ribbon shaped particles of variable length sometimes exceeding 300 nm. The in-house (non-commercial) materials, comprising mostly of rod shaped nanoparticles with an average length of *ca.* 40–60 nm, slightly varied in the level of roundness, with the ones of nHA-HC being more elongated than that of nHA-LC. This variation may have originated from the preparation method and/or could have been a side effect of the scaling up process of material production.

Interestingly, it was observed that the resulting materials (nHA-LC and nHA-HC) also had different consistencies and appearance: nHA-LC was more paste like, while nHA-HC more like a transparent gel. It was possible to concentrate the nHA-HC produced on a small scale up to nearly 50 wt% while preserving its injectability properties, whereas nHA-HC, produced at large scale, was hard to concentrate above *ca.* 40% as increasing the ceramic content led to a loss of its fluidic properties. It is out of the scope of this manuscript to discuss the differences in the rheological behaviour and optical properties of the pastes and their possible relation to a scale up process, and therefore this manuscript will focus on the *in vivo* performance of the materials.

### Radiography results (x-ray, micro-CT)

**Figure 1:**
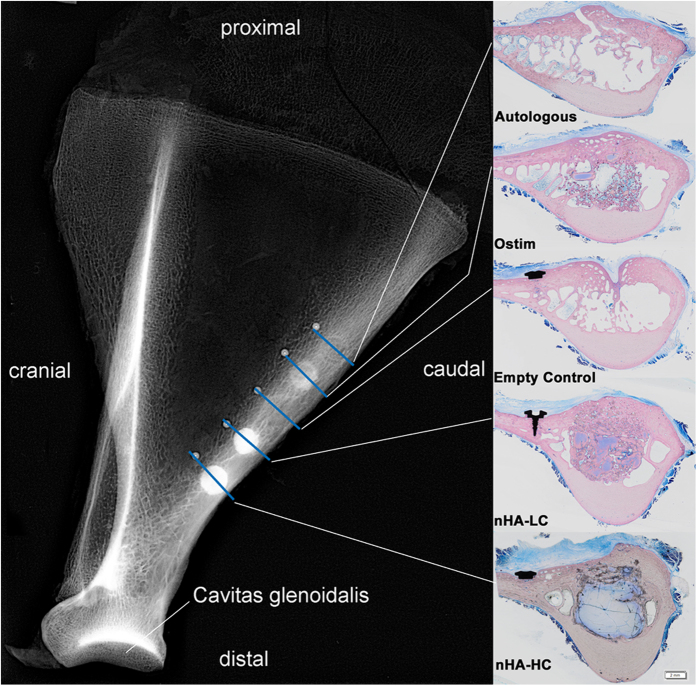
Representative example of the surgical site with marks for the histological sectioning (X-ray, 60 kV, 0.32 s, 6 months after augmentation): Five defects were placed at the caudal edge of the left scapula and filled in a randomised order with the 4 test substances while 1 was left empty. Every defect site was marked with a titanium pin. Images of the corresponding histological sections, stained with Levai-Laczko dye, are depicted on the right.

**Figure 2:**
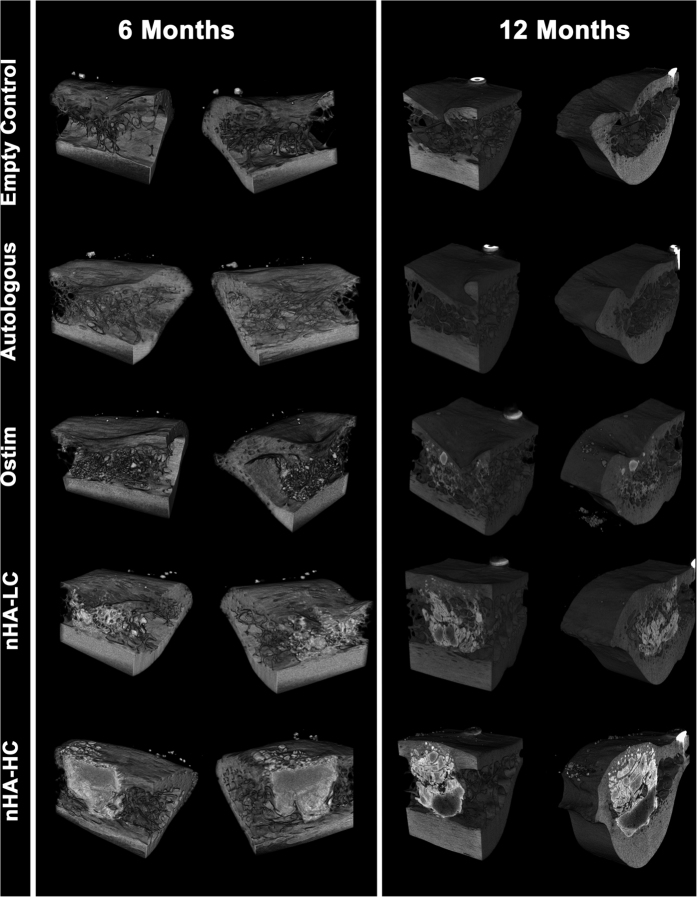
Micro-CT reconstructions of representative defect sites 6 months (left) and 12 months (right) after implantation. Defects left empty or filled with autologous bone or Ostim^®^ showed a non-bridging or cavity formation in the cortical region. nHA-LC and nHA-HC were able to bridge the defect site at the cortical area. A high amount of dense nHA-HC was found in the defect site.

Animals were sacrificed at 6 months or 12 months after surgery. The x-ray images of the defects filled with the HA pastes Ostim^®^, nHA-LC and nHA-HC indicated that material was present in the defect area at both time points. The defect areas of the control group and of the autologous group were detectable after 6 and 12 months on x-ray images (Fig. 1). The reconstruction of the micro-CT evaluation after 6 months showed a non-bridging or cavity formation in the cortical area of the defects in all groups except the defects filled with nHA-HC (Fig. 2). After 12 months the control group, the autologous group and the defects filled with Ostim^®^ showed a non-bridging or cavity formation at the defect sites, whereas the groups nHA-LC and nHA-HC showed a complete reconstruction of the cortical bone (Fig. 2). nHA-LC, nHA-HC and Ostim^®^ were detectable in the micro-CT at both time points. The pastes, nHA-LC and Ostim^®^ separated into particle units or agglomerates, whereas nHA-HC remained mainly compact in the form of a single large agglomerate and showed bone integration only in the periphery of this structure (Fig. 2).

After 6 months, 27% of the empty control group (3 out of 11), 17% of the autologous group (2 out of 12) and 55% of the Ostim^®^ filled defects (6 out of 11) presented a bridging, whereas 82% of the nHA-LC group (9 out of 11) and 92% of the nHA-HC filled defects (11 out of 12) showed a bridging of the defect. After 12 months, bridging was detectable for 33% for the empty control group (4 out of 12), 25% for the autologous group (3 out of 12), 42% for the Ostim^®^ filled defects (5 out of 12), but for 75% of the nHA-LC group (9 out of 12) and 83% for the nHA-HC filled defects (10 out of 12).

### Histologic evaluation

The margins of the drill holes and their original size were readily detectable in the histologic specimens (see Supplementary Fig. 4). The existing bone present at the edges of the defect stained a lighter shade of pink compared to the new bone tissue that had regenerated inside of the drill hole, which was darker and purple in colour. There were distinct differences between different regions of the defect area. While in the medullary compartment cancellous bone tissue was formed in an amount and in a quality that was comparable to the pre-surgical state, in the cortical area bone regeneration was usually impaired. In the control group which received no bone substitute material at all, deep concave depressions were visible in the cortical bone. Additionally, in many cases the bony cortex was not completely continuous but was breached by canals that connected the bone marrow with the surrounding periosteal region, i.e. the defects were not completely bridged. This indicates that in the chosen animal model no final defect healing is achieved spontaneously, neither after 6 nor after 12 month. Even when treated with autologous bone, depressions, canals and gaps were present in the cortical layer. Only very small remnants of the bone graft were still detectable in the augmented regions.

The three tested HA-pastes exhibited very different results in several aspects. The most obvious was the amount, size and size variation of the remaining agglomerates of HA-paste. In the Ostim^®^ group many of these agglomerations formed numerous particles that had about the size of an osteon (200 μm). In the nHA-LC group most of the agglomeration particles were of about the same size, but there were also a few much larger particles, often more than 5 mm in diameter. This trend was even more pronounced in the nHA-HC group where single particle agglomerations, significantly larger than in the nHA-LC group, were detectable, often filling the whole defect.

Concerning the regeneration of the cortical layer, Ostim^®^ and nHA-LC exhibited results that were comparable to the control groups. The cortical areas showed depressions and canals connecting the marrow space with the soft tissue surrounding the scapula although not as severe and frequent as in the controls. In the nHA-HC group on the other hand the cortical bone overlaying the large agglomerations was more continuous, depressions less pronounced or absent and the prevalence of holes in the compact bone lower.

No great differences were seen in the osteoconductive properties of the bone substitutes. Most of the surfaces of all three materials were covered with thin layers of vital, newly formed bone, demonstrating their ability to enhance and promote bone regeneration. On those parts of the surfaces not covered with bone, very often signs of resorption could be seen. These occurred in the form of resorbing, multinucleated cells, resorption lacunae and more often discoloured seams that are characteristic of the cellular degradation of bone substitute materials.

Histological findings changed comparatively little between 6 and 12 months. Most prominent is the reduction of the size of particle agglomerations also indicating that resorption of HA-pastes is continuously proceeding over considerable periods of time.

### Histomorphometric evaluation

#### Bone Substitute Volume/Tissue Volume

**Figure 3:**
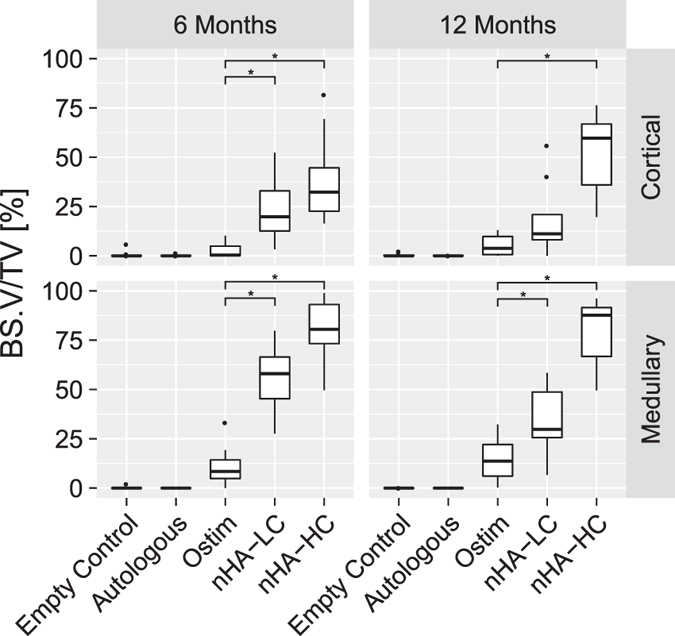
Bone substitute volume (BS.V.) per tissue volume (TV): percentage of the regions of interest that are filled with bone substitute material. Among the three HA-paste fillings, nHA-HC showed the highest and Ostim^®^ the lowest BS.V/TV ratios at both time points in both areas of interest (*p-value < 0.05 compared to Ostim^®^).

The BS.V/TV describes the percentage of bone substitute material in the cortical or medullary defect areas. Compared to Ostim^®^, a statistically significant increase in BS.V/TV was detected at 6 months in both regions of interest for nHA-LC (p < 0.001) as well as nHA-HC (p < 0.001). At 12 months, the BS.V/TV was increased in the medullary region for nHA-LC (p < 0.001) and nHA-HC (p < 0.001) compared to Ostim^®^. In the cortical region at 12 months elevated levels of BS.V/TV of defects filled with nHA-HC were detected compared to Ostim^®^ (p < 0.001) and nHA-LC (p < 0.001) (Fig. 3).

##### Number and size of bone substitute particles

**Figure 4:**
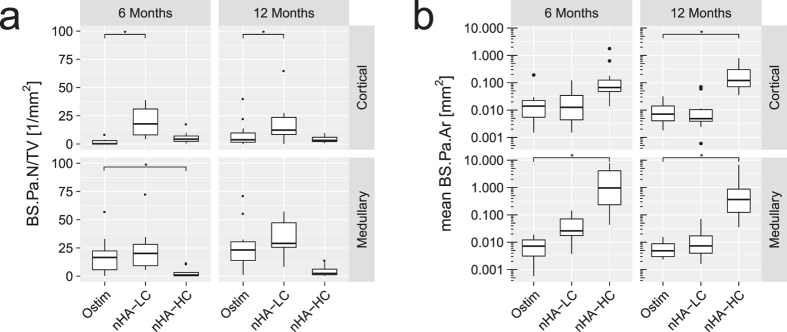
(**a**) Number of bone substitute particle agglomerates (BS.Pa.N) per tissue volume (TV): In the cortical region, an increase in nHA-LC BS.Pa.N/TV was detected. In the medullary region at 6 months, a decrease in nHA-HC BS.Pa.N/TV was measured. (**b**) Average size of bone substitute particle agglomerates (BS.Pa.Ar) as measured in the region of interest in mm^2^ presented on a logarithmic scale. nHA-HC showed an increased agglomerate size with means ranging from 0.22 to 2.67 mm^2^, compared to the mean agglomerate sizes of nHA-LC and Ostim^®^ ranging below 0.051 mm^2^ (*p-value < 0.05 compared to Ostim^®^).

The nanoparticles of the three different pastes formed larger particle agglomerates, their size and number could then be determined on the histological sections. In the cortical region, nHA-LC showed the highest BS. Pa.N. per mm^2^ compared to Ostim^®^ (p < 0.001 at 6 months, p < 0.015 at 12 months) and nHA-HC (p < 0.001 at 6 and 12 months). In the medullary region at 6 months, a significant decrease of nHA-HC BS.Pa.N/TV was found compared to Ostim^®^ (p < 0.033) and nHA-LC (p < 0.003). At 12 months, a decreased amount of nHA-HC BS.Pa.N/TV was measured compared to nHA-LC (p0.001) (Fig. 4a).

The BS.Pa.Ar. of all pastes showed a tendency to decrease from month 6 to month 12, indicating a certain break-down and resorption of the material (Fig. 4b). At 6 months, no significant difference in BS.Pa.Ar. could be defected in the cortical region. In the medullary region an increased nHA-HC particle agglomerate size was detected compared to Ostim^®^ (p < 0.001) and nHA-LC (p < 0.001). At 12 months, the BS.Pa.Ar. of nHA-HC in the cortical and medullary region was found to be significantly increased compared to Ostim^®^ (p < 0.001 and p = 0.026, respectively) and nHA-LC (p < 0.001 and p = 0.028, respectively) (Fig. 4b).

#### Newly formed bone volume and the composite volume of bone substitute and newly formed bone in the regions of interest

**Figure 5:**
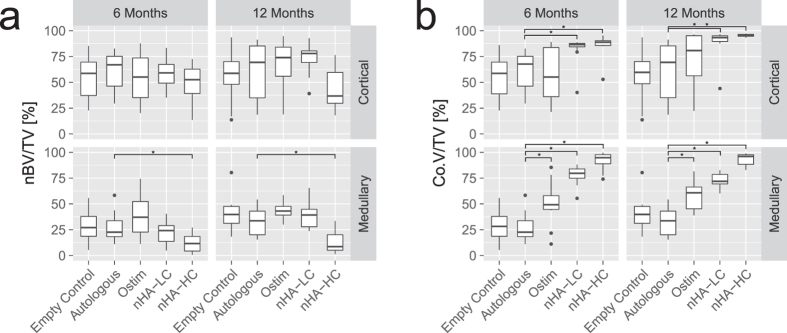
(**a**) Newly formed bone volume (nBV) per tissue volume (TV): Comparable results were found between the defect fillings at both regions of interest and both time points, except the defects filled with nHA-HC, which showed a lower bone bone volume per TV in the medullary region. (**b**) Defects filled with Ostim^®^, nHA-LC and nHA-HC showed an increased composite volume (Co.V) per tissue volume (TV) compared to the autologous group at both time points in the medullary region (p < 0.001). In the cortical region, a significant increase at both time points in Co.V/TV between defects filled with nHA-LC and nHA-HC and the autologous group (p ≤ 0.018) were found (*p-value < 0.05 compared to autologous control).

At 6 months, no significant differences in nBV/TV were measured in the cortical region. In the medullary region a reduced amount of nBV/TV was detected for the defects filled with nHA-HC compared to the empty control (p = 0.018), the autologous bone (p = 0.038) and Ostim^®^ (p < 0.001). Also between the defects treated with Ostim^®^ and nHA-LC a significant difference was found (p = 0.017) (Fig. 5a). At 12 months, a significant decrease in nBV/TV in the cortical region between defects augmented with nHA-LC and nHA-HC was detected (p = 0.011). In the medullary region defects filled with nHA-HC showed a decrease in nBV/TV compared to all other groups (p < 0.001) (Fig. 5a).

The highest amount of composite volume, which is the sum of volumes of newly formed bone and bone substitute material, per TV was found at both time points and in both areas of interest in the defects filled with nHA-HC. Up to 97.1% (12 months, cortical region) of the region of interest treated with nHA-HC consisted of composite material (Fig. 5b). Defects filled with nHA-LC showed Co.V/ TV means in the cortical region of 82.1% (month 6) and 88.8% (month 12); and in the medullary region of 77.6% (month 6) and 73.1% (month 12). Defects augmented with Ostim^®^ resulted in Co.V/TV means in the cortical region of 56.8% (month 6) and 71.4% (month 12) and in the medullary region of 49.9% (month 6) and 58.6% (month 12) (Fig. 5b).

#### Soft tissue ingrowth-Periosteal void volume per TV

**Figure 6:**
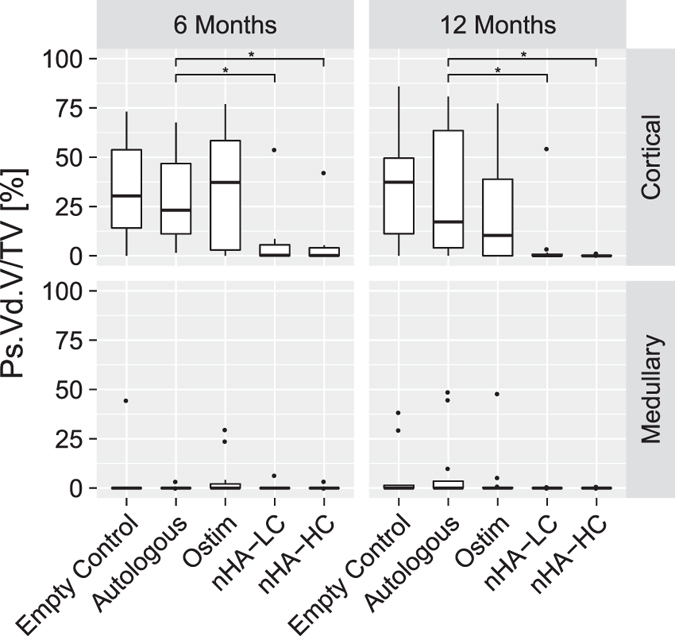
Periosteal void volume (Ps.Vd.V) per tissue volume (TV) presenting the soft tissue ingrowth into the defect site. Very limited soft tissue ingrowth was detected in the medullar region for all treatment groups and at both time points. In contrast, soft tissue ingrowth was found in the cortical region when the defects were filled with Ostim^®^, autologous bone or left empty. nHA-HC and nHA-LC showed a decreased soft tissue ingrowth at both time points (*p-value < 0.05 compared to autologous control).

The PS.Vd.V/TV shows the percentage of soft tissue that has penetrated from the periosteal region into the defect area. In general, more soft tissue ingrowth was found in the cortical region compared to the medullary region. No significant difference was found in the medullary region between the groups at both time points. In the cortical region at 6 months, a significant decrease of soft tissue ingrowth was found for the defects filled with nHA-LC compared to the empty control group (p = 0.004), the autologous group (p = 0.034) and Ostim^®^ (p = 0.006). Similarly, the defects augmented with nHA-HC showed a significant decrease in soft tissue ingrowth compared to the empty control (p = 0.002), the autologous group (p = 0.019) and Ostim^®^ (p = 0.003). Also after 12 months, less soft tissue ingrowth was observed for defects filled with nHA-LC compared to the empty control (p = 0.001) and the autologous group (p = 0.010). Defects filled with nHA-HC showed a decreased soft tissue ingrowth compared to the empty control (p < 0.001), the autologous group (p = 0.001) and Ostim^®^ (p = 0.040) (Fig. 6). No significant differences were detected between Ostim^®^ and the control groups or between nHA-LC and nHA-HC.

A summary of the histomorphometric evaluation can be found in Supplementary Table 1 (cortical region) and Supplementary Table 2 (medullary region).

## Discussion

It has been reported that sheep present a valuable model for human bone turnover and remodelling. It was demonstrated that the ovine bone ingrowth and mineral acquisition into porous-coated implants was comparable to that of humans^29^. Additionally, the ovine weight and bone dimensions are more similar to humans than other animal models and furthermore, aged sheep have been also shown as suitable model for human osteopenic and osteoporotic bone^30^,^31^. While the x-ray evaluation of the bones confirmed that the defects were placed at the same locations of the left scapula, the micro-CT showed that only the upper cortical bone and medullary regions were involved, whereas the lower cortical region remained intact. The empty defects resulted in non-bridging (average of 70% of the defects) or cavity formation, suggesting that an adequate model and time points were chosen.

HA is widely used in the clinics and has been reported as a biocompatible and osteoconductive bone substitute material^8^,^9^,^11^,^14^,^32^,^33^. Ostim^®^, a nanoparticular HA aqueous paste, has been described as bone-inducible paste. Several publications observed that Ostim^®^ particles were not fully resorbed 12 weeks post-surgery^9^,^11^,^12^,^34^. In general all HA pastes used in this study were shown to support bone formation. 12 months post-surgery the three HA pastes were, to varying degrees, detectable in all defects. The HA content of the pastes related to the amount of BS.V/TV, and Co.V/TV. However, as the particle size and shape also varies among the three pastes, the performance of the pastes cannot be merely attributed to their HA content. We observed nanoparticle agglomerates of various sizes for all three pastes. nHA-HC agglomerates presented mean sizes ranging from 0.22 mm^2^ to 2.67 mm^2^. In the medullary region, nHA-LC agglomerates showed mean sizes of 0.051 mm^2^ after 6 months and 0.017 mm^2^ after 12 months. The mean agglomerate size of Ostim^®^ in the medullary region was 0.008 mm^2^ after 6 months and 0.006 mm^2^ after 12 months. These differences in agglomeration behaviour may explain the significantly quicker resorption of Ostim^®^ compared to nHA-LC that has a similar HA content. The BS.Pa.N/TV evaluation underlined the particle aggregation of the nHA-HC paste resulting in a very low particle number but large particle size. The trend of BS.Pa.N/TV increase along with the tendency of a mean BS.Pa.Ar. decrease for nHA-LC and Ostim^®^ in the medullary region from month 6 to 12 indicated the break-down of the particle agglomerates into smaller agglomerate subunits. The defects filled with Ostim^®^ and autologous bone resulted in a cavity formation and a low ratio of bridging defects (average of 48% for Ostim^®^ and 21% for autologous bone). In contrast, defects filled with nHA-LC and nHA-HC showed a cortical bone formation with an average bridging ratio of 78% (nHA-LC) and 88% (nHA-HC), respectively. This effect was observed in the micro-CT evaluation and confirmed by the Ps.Vd.V/TV results, showing soft tissue ingrowth in the defect sites. Dau *et al*. created mono cortical critical size bone defects in minipigs that were filled with the two nanoparticular HA pastes Ostim^®^, NanoBone^®^ or the natural bone substitute material Bio-Oss^®^, which is derived from the mineral portion of bovine bone. While after 8 months, Ostim^®^ showed the least amount of residual material at the defect sites, no differences in new bone formation or soft tissue ingrowth was detected between the tested groups. They recommended NanoBone^®^ or Bio-Oss^®^ for use in defects that require stability of the bone^35^. In contrast to Dau *et al*., our study detected an increased soft tissue ingrowth in the Ostim^®^ group compared to the other HA pastes. Based on our findings nHA-LC should be chosen for stable bone integration.

Interestingly, autologous bone grafts failed to improve bone formation or decrease soft tissue ingrowth, giving comparable results to the empty control. Bone grafts undergo extensive remodelling and resorption during the first year. Dependent on the nature of the grafted material and implantation site, various autologous bone resorption rates have been reported^36^. A 29% resorption rate of autologous bone grafts from the iliac crests used for sinus floor augmentation was observed after 3 months^37^. Van der Meij *et al*. reported the presence of approximately 30% of autologous bone graft from the iliac crest for cleft lip and palate treatment after 1 year^38^. Autologous bone blocks for horizontal ridge augmentation lost 28% of its volume during 6 months^39^. In our study a low amount of the autologous cancellous bone was detectable at both time points, indicating a fast resorption of the grafted material. The fast resorption of the autologous bone graft may explain the similar results to the empty defects. Several publications reported a size-dependent cytotoxic effect of nanoparticles *in vitro* and *in vivo* when administering particle suspensions^40^,^41^,^42^,^43^. Clinical studies evaluating the suitability of Ostim^®^ as a bone-filling matrix reported good bone induction with limited or no side effects^44^,^45^,^46^,^47^,^48^. Huber *et al*. reported that biopsy analysis showed good bone formation without inflammatory reactions, osteofibrosis or osteonecrosis^48^. In this study we observed good bone regeneration for all three HA pastes without any inflammatory reaction, osteofibrosis or osteonecrosis. The liquid phase of the water-based HA pastes can decrease over time, which could explain the formation of dense particle agglomerates^11^. Therefore, the previous studies evaluating monodisperse particle solution might not represent the observed *in vivo* paste behaviour.

In this study we observed a strong correlation between HA content, the size of the particle agglomerates with the amount of newly formed bone tissue and defect bridging. While a high HA content of 48 wt% with larger particle agglomerates supported defect bridging, less newly formed bone tissue was obtained within the defect site. This is probably caused by the fact that large particle agglomerates leave less space for the formation of new bone. On the other side, a lower HA content of 35 wt% (Ostim^®^) and 38 wt% (nHA-LC), respectively, was correlated with smaller particle agglomerates, an increased amount of newly formed bone tissue within the defect but also a reduced defect bridging ratio. The impact of particle agglomeration behaviour must be examined in more detail in further studies.

## Conclusion

In this preclinical study, three bone graft pastes of different HA concentrations and particle sizes were compared to treatment of defects with autologous bone and empty defects. Bone regeneration and growth but incomplete defect healing was observed in the empty control group and by treatment with autologous bone. However, a cavity at the defect site was visible. While the higher concentrated (48 wt%) nHA-HC agglomerated and supported bone formation only on the outer surface of the material, Ostim^®^ (35 wt%) degraded the quickest and could not fill the defect for a sufficient period of time to allow for a complete cortical bone bridging. This was similar to the autologous bone filling where a cavity at the defect site, an increase in soft tissue ingrowth and a low bridging rate were observed. We suggest that the agglomeration behaviour presents a critical parameter for nanoparticular HA pastes due to their influence on material resorption and bone formation. nHA-LC (38 wt%) seemed to have the best combination of characteristics that lead to good bone regeneration, reduced soft tissue ingrowth, had a high defect bridging rate and optimal particle agglomeration size, while keeping in mind the limitations of animal studies.

## Materials and Methods

### Materials preparation and characterisation

Commercially available Ostim^®^ was obtained from Heraeus Kulzer, Germany. Nano-hydroxyapatite (nHA) was prepared as previously described^49^. In brief, aqueous solutions of calcium chloride and potassium phosphate monohydrate were fed into the inlets of either large or small scale NETmix reactors at a molar ratio of 1.67, or 5:3^50^, under controlled pH of 11–12 using potassium hydroxide (all reagents from Sigma-Aldrich, Spain). Resultant slurries were further concentrated to viscous pastes/gels of *ca.* 38 and 48 wt% and were given codes nHA-LC for material prepared in a large scale reactor and nHA-HC for material from a small scale reactor, respectively.

Thermogravimetric analysis (TGA) was performed on nHA-LC and nHA-HC materials to quantify the concentration of water and ceramics (nHA) in the systems. TGA was carried out using a PerkinElmer Pyris 1 thermogravimetric analyser. Heating was performed in a platinum crucible in nitrogen flow (20 cm^3^·min^−1^) at a rate of 10 °C min^−1^ from 30 °C up to 1000 °C. Proportions of the materials were dried at 60 °C and further ground for performing phase analysis using x-ray powder diffraction (XRPD) method.

Aliquots of the pastes as well as Ostim^®^ were dissolved in distilled water and dispersed onto a holey carbon film supported by a 300 mesh copper grid for particle size evaluation using a transmission electron microscope (TEM) JEOL JEM-3010 with a LaB_6_ electron gun at an accelerating voltage of 300 kV.

X-ray powder diffraction (XRPD) patterns were acquired using a Siemens D5000 Diffractometer operating at 40 kV and 40 mA with Cu Kα radiation. Analysis of the obtained patterns was carried out using X’Pert HighScore Plus software, and stripping of the Kα-2 component was carried out when appropriate.

### Ethical conduct of research

The study was approved by the local Thuringia State Office ethical committee (“Thüringer Landesamt für Verbraucherschutz”) and was conducted in accordance with the principles of the 1964 Declaration of Helsinki.

### Animals and anaesthesia

For this study, 24 adult (age of 2-3 years) female land merino sheep that during their life had given birth at least 1-2 times were housed in a freestall barn, fed with hay and allowed to drink water *ad libitum*. The study was approved by the local ethical committee for animal experiments (see chapter Ethical conduct of research). Haematological and parasitological examinations were performed prior to the study. Animals were operated in two groups in the late autumn. General anaesthesia was induced by an intravenous administration of 0.1 mg/kg Diazepam, 4.4 mg/kg Ketamin and 0.1 mg/kg Xylazin. After induction, the animals were intubated and connected to a semi-open anaesthesia system with an isoflurane concentration of 1.0–1.5 volume% and an oxygen supply of 2 l O_2_/min. The animals received a pre-operative systemic antibiotic (3 ml/50 kg Veracin-Compositum i.m.) as well as the analgeticum Rimadyl^®^ (Pfizer, 1 ml/35 kg i.v.).

### Surgical procedure, material application and post-operative medication

A longitudinal incision along the caudal border of the left scapula was made and the border was exposed by sharp dissection. Using a trephine drill, five defects with a diameter and depth of 8 mm were created, leaving a 5 mm bony bridge in between each defect^51^. The longitudinal axis of the drill holes was oriented perpendicular to the lateral face of the scapula. The bony cylinders from the drill holes were preserved for autologous bone grafting. Titanium pins were placed close to the periphery of the holes to mark the exact location of the defect areas. One defect remained empty and served as a control. The other defects were filled with one of four materials: (1) autologous bone graft, (2) the commercially available Ostim^®^ with a HA content of 35 wt%, (3) injectable paste with a concentration of 38 wt% HA-crystals (nHA-LC) and (4) paste with a high concentration of 48 wt% HA-crystals (nHA-HC) (Fig. 1). The five groups were randomised to the defect sites for each animal prior to the procedures. The surgical site was closed in multiple layers using Monocryl 3-0 suture material. The animals received Rimadyl^®^ 1 ml/35 kg s.c. five days post-operatively. Twelve animals were euthanised after either 6 or 12 months by the administration of T 61^®^ i.v.

### Histology and sample preparation

The scapulae were removed and fixed in buffered formalin. Overview radiographic images were produced to ascertain the exact location of the augmentation sites. Blocks, each of them containing one of the defects were dissected using a diamond coated band saw (EXAKT Apparatebau, Norderstedt, Germany) by cutting precisely in between the drill holes as visible on the radiograph. These blocks were subjected to micro-CT (μCT) analysis (see section “Micro-CT analysis”). After the μCT scan the specimen blocks were dehydrated, embedded in plastic and then undecalcified thin sections were produced using the method of Donath^52^. Histologic specimens were stained with Levai-Laczko dye and photographed with an Olympus BX61VS scanning microscope (Olympus DotSlide system 2.4; Olympus, Tokyo, Japan) at a resolution of 0.321 μm/pixel.

### Micro-CT analysis

The samples were scanned in a SCANCO μCT 50 at 90 kV with an isotropic resolution of 17.2 μm. The scans were exported as 16 bit DICOM image stacks. Based on the micro-CT evaluation the closure of the defects was categorised as “non-bridging” (leaving a continuous opening in the cortical bone connecting the medullary cavity with the periosteal area) or “bridging” when the continuity of the cortical bone was completely restored and no opening remained.

### Histomorphometric analyses

The histological images were downsampled to 25% (1.283 μm/pxl) and then automatically segmented using Definiens Developer XD 2.1 (Definiens AG, Munich, Germany). Errors in segmented images were corrected manually using Photoshop CS 4 (Adobe Systems Inc., San Jose, CA, USA). The corrected images were then measured in Definiens Developer XD 2.1. A quadtree segmentation, in which square objects are quartered until they fulfil set homogeneity criteria, was applied to the images. The resulting square objects were first classified in a very rough manner to determine the colour balance of the individual image. The background (soft tissue) was classified using a very conservative threshold and then grown into surrounding background using a less restrictive threshold. Bone in the remaining area was classified using differences in the colour layers. Bone objects which were not bordered by other bone objects on at least two sides were declassified to remove potential misclassified bone objects. In the resulting segmented bone, the mean difference between the red and green layers were measured and used to calculate the threshold for a more accurate segmentation. Bone and the unclassified area around it was thresholded using this calculated threshold on the pixel level. The border between bone and background was smoothed using alternating pixel based growing and shrinking constrained by surface tension. Small objects completely surrounded by another class were added to the surrounding class. The border was further improved by allowing it to shift position slightly if this increased the difference between the separated classes in the area. The segmentation was exported for manual correction. The regions of interest were selected before the correction. The cortical region of interest was defined as the area between the boundaries of the drill hole in the cortical bone and the periosteal and endosteal margins. The medullary region of interest was located 1 mm beneath the cortical region of interest, was oriented parallel to it and had a height of 1 mm. The medullary region of interest also spanned the whole breadth of the drill hole and lay solidly within the marrow space.

As the substitute materials could not be reliably distinguished automatically these were classified entirely manually. The corrected images were then measured in Definiens. The areas of newly formed bone (nBV), background or soft tissue (Vd.V), substitute material (BS.V) and the complete region of interest (TV; tissue volume) were measured. Also the sizes of the individual particle agglomerates (BS.Pa.V) and their number (BS.Pa.N) were determined. From these primary measurements the following parameters were calculated: The percentage of bone substitute material in the regions of interest (BS.V/TV), the average size of particle agglomerates (BS.Pa.Ar) in mm^2^, the number of bone substitute particle agglomerates per mm^2^ (BS.Pa.N/TV), the percentage of newly formed bone in the regions of interest (nBV/TV), the percentage of the composite of newly formed bone plus bone substitute material (Co.V/TV), the percentage of the region of interest filled with soft tissue that had grown from the periosteal region into the defect area (Ps.Vd.V; Periosteal void volume).

### Statistical analysis

All animals were included in the final analysis; one defect site (6 months, Ostim^®^) was excluded due to faulty drilling. Two other defects (6 months, empty control and autologous) were excluded from the bridging/non-bridging analysis due to later micro-CT reconstruction problems. For inference, multiple linear mixed models for all independent variables were built using treatment as independent variable and animal ID as a random factor. We then proceeded to calculate Tukey-type post-hoc tests for treatment effect^53^. All computations were done using R version 3.2.3^54^. Statistical Graphs were created using ggplot2^55^.

## Additional Information

**How to cite this article:** Hruschka, V. *et al*. Comparison of nanoparticular hydroxyapatite pastes of different particle content and size in a novel scapula defect model. *Sci. Rep.* **7**, 43425; doi: 10.1038/srep43425 (2017).

**Publisher's note:** Springer Nature remains neutral with regard to jurisdictional claims in published maps and institutional affiliations.

## Supplementary Material

Supplementary Information
