# Supplementary material for: Comparison of nanoparticular hydroxyapatite pastes of different particle content and size in a novel scapula defect model
Source: Sci Rep. 2017 Feb 24;7:43425. doi: 10.1038/srep43425 (PMC5324075; doi:10.1038/srep43425)
Supplement: Supplementary Information [file srep43425-s1.pdf]

Title: **Comparison of nanoparticular hydroxyapatite pastes of different particle content and size in a novel scapula defect model.**

Authors: Veronika Hruschka*,[1,3], Stefan Tangl[2,3], Yulia Ryabenkova[4], Patrick Heimel[1,2,3], Dirk Barnewitz[5], Günter Möbus[4], Claudia Keibl[1,3], James Ferguson[1,3], Paulo Quadros[6], Cheryl Miller[7], Rebecca Goodchild[8], Wayne Austin[8], Heinz Redl[1,3], Thomas Nau[1,3]

Supplementary Figures

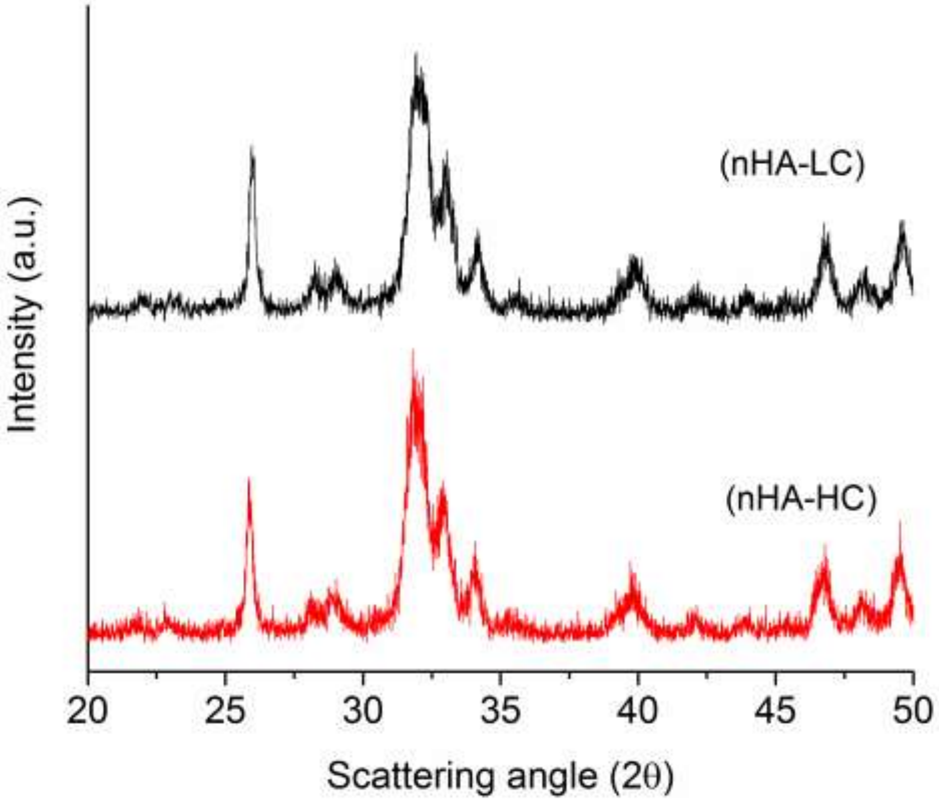

Supplementary Figure 1: XRPD patterns of nHA-LC (top) and nHA-HC (bottom)

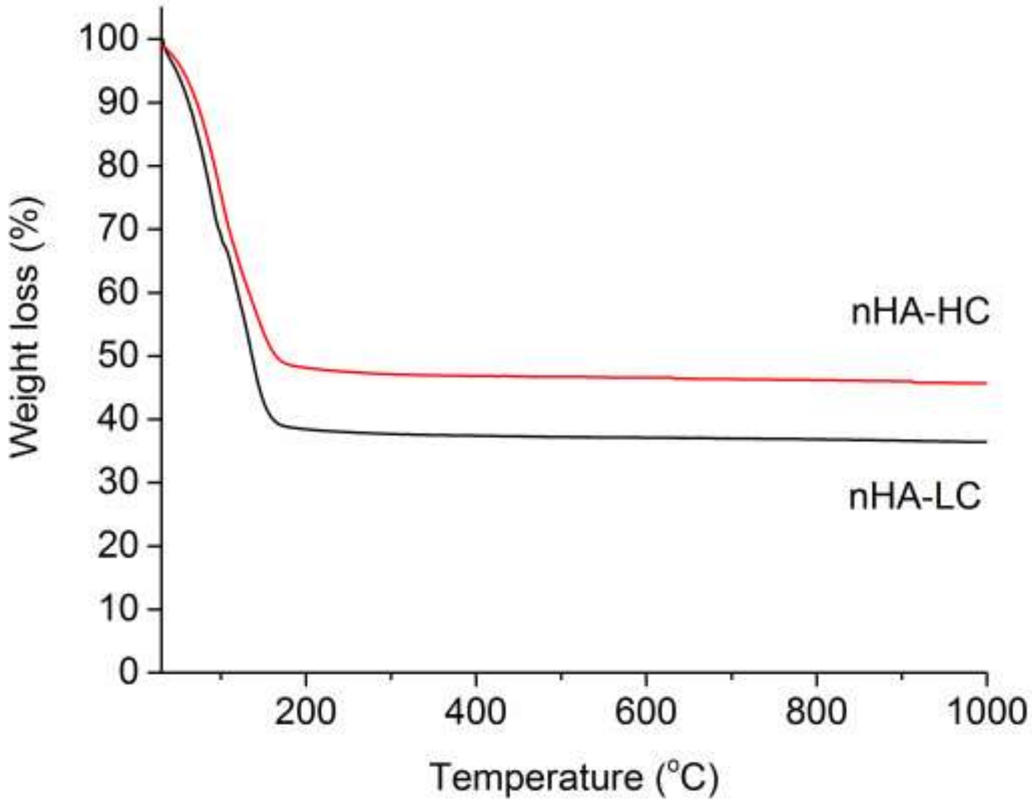

Supplementary Figure 2: Thermogravimetric analysis of nHA-LC and nHA-HC

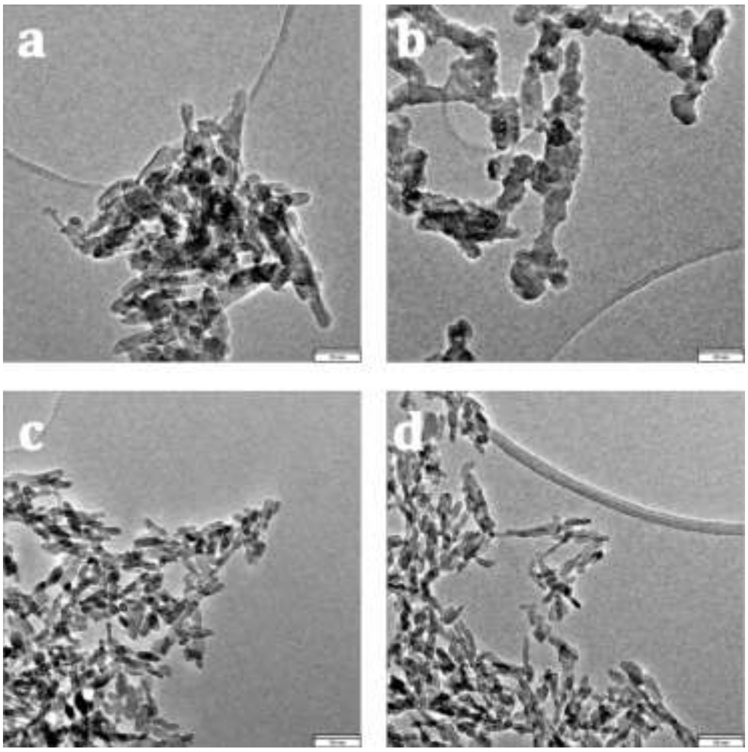

Supplementary Figure 3: Bright-field TEM micrographs of Ostim (a, b), nHA-LC (c) and nHA-HC (d). Scale bar 70 nm.

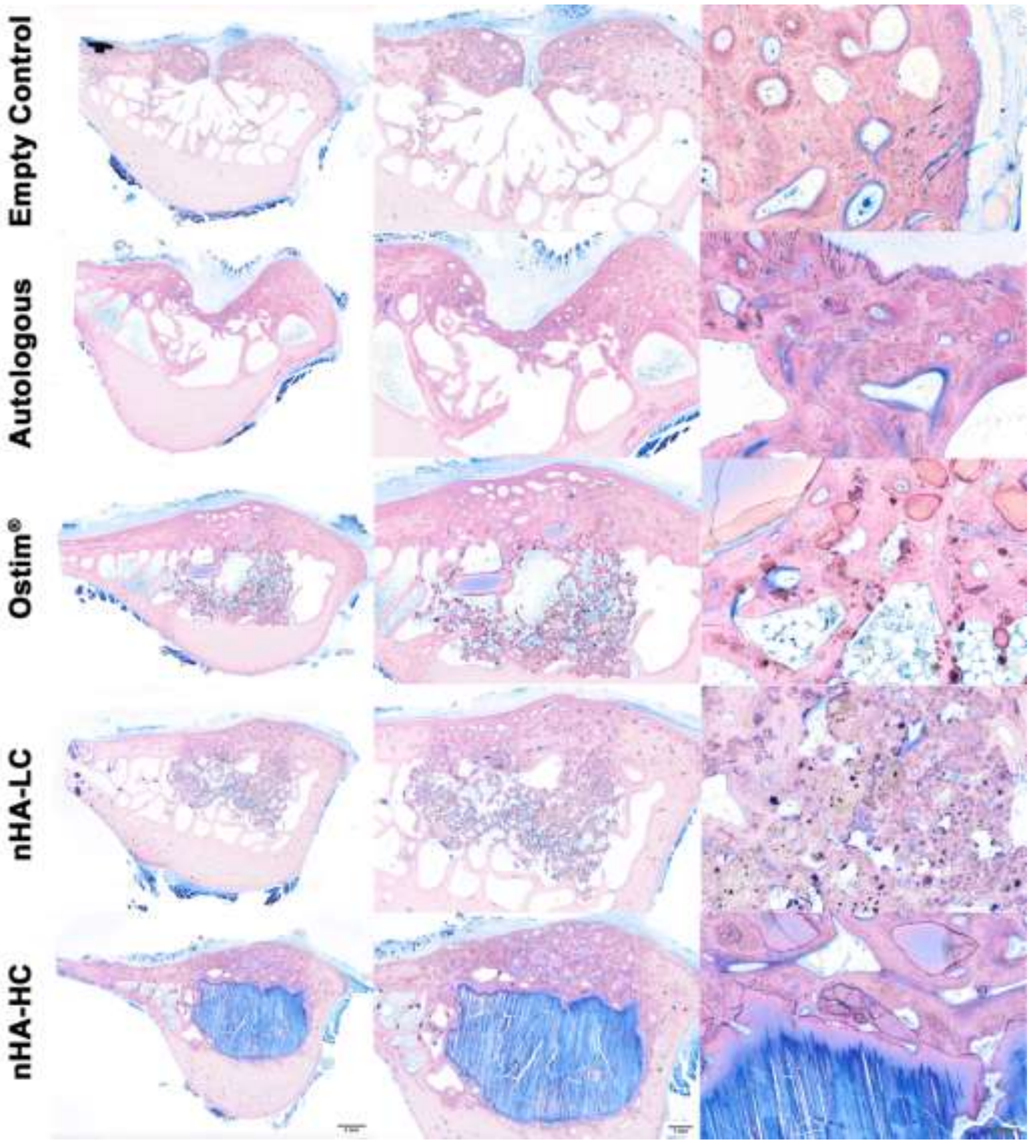

Supplementary Figure 4: Undecalcified thin ground sections, stained with Levai-Laczko dye. Incomplete bridging of the cortical area in the empty control group. Concave depression in the cortical area of the autologous bone group. Defects are filled with particle agglomerates and surrounding newly formed bone in all groups treated with HA-pastes. The nHA-HC group shows particle agglomerates of very large size.

Cortical Region

		BS.V/TV	BS.Pa.N/TV	BS.Pa.Ar. [mm²]	nBV/TV	Co.V/ TV	PS.Vd.V/TV	Bridging rate
**Empty Control**	**6 months**	0.57	-	-	54.39	54.96	**35.06**	27%
**Autologous**		0.13	-	-	**60.36**	60.49	29.72	17%
**Ostim**		2.90	1.77	0.027	53.86	56.77	34.53	55%
**nHA-LC**		23.10	**19.50**	0.024	58.98	82.09	6.53	82%
**nHA-HC**		**37.52**	5.40	**0.258**	48.36	**85.88**	4.93	**92%**
**Empty Control**	**12 months**	0.35	-	-	56.34	56.69	**37.26**	33%
**Autologous**		0.03	-	-	59.83	59.87	32.67	25%
**Ostim**		5.04	8.52	0.010	66.32	71.36	23.97	42%
**nHA-LC**		16.97	**18.40**	0.015	**71.87**	88.84	5.03	75%
**nHA-HC**		**51.78**	4.19	**0.220**	43.77	**95.55**	0.15	**83%**

Supplementary Table 1: Mean values of bone subistute volume (BS.V) per tissue volume (TV), bone substitute particle number per TV, average size of bone substitute particle agglomerates (BS.Pa.Ar), newly formed bone volume (nBV) per TV, composite volume (Co.V) per TV, periosteal void volume (PS.Vd.V) per TV and defect bridging rate in the cortical region. The highest value per parameter is marked bold.

Medullary Region

		BS.V/TV	BS.Pa.N/TV	BS.Pa.Ar. [mm²]	nBV/TV	Co.V/ TV	PS.Vd.V/TV
**Empty Control**	**6 months**	0.18	-	-	28.52	28.70	3.71
**Autologous**		0.00	-	-	27.16	27.16	0.28
**Ostim**		10.82	18.06	0.008	**39.05**	49.88	**5.24**
**nHA-LC**		55.37	**22.68**	0.051	22.23	77.60	0.54
**nHA-HC**		**79.95**	2.92	**2.672**	11.81	**91.76**	0.28
**Empty Control**	**12 months**	0.03	-	-	40.56	40.59	5.92
**Autologous**		0.00	-	-	32.41	32.41	**8.75**
**Ostim**		14.84	25.69	0.006	**43.73**	58.56	4.51
**nHA-LC**		34.35	**47.24**	0.017	38.78	73.13	0.05
**nHA-HC**		**80.28**	4.57	**1.258**	12.64	**92.92**	0.07

Supplementary Table 2: Mean values of bone subistute volume (BS.V) per tissue volume (TV), bone substitute particle number per TV, average size of bone substitute particle agglomerates (BS.Pa.Ar), newly formed bone volume (nBV) per TV, composite volume (Co.V) per TV, periosteal void volume (PS.Vd.V) per TV in the medullary region. The highest value per parameter is marked bold.
